# The Role of the Built Environment on the Quality of Life for Residents in Long-Term Care Facilities in Asia: A Scoping Review

**DOI:** 10.1093/geroni/igac045

**Published:** 2022-07-01

**Authors:** Habib Chaudhury, Mingjun Xu

**Affiliations:** Department of Gerontology, Simon Fraser University, Vancouver, British Columbia, Canada; School of Geography and Planning, Sun Yat-sen University, Guangzhou, Guangdong, China

**Keywords:** Design, Functioning, Nursing home

## Abstract

**Background and Objectives:**

The quality of the built environmental features in long-term care (LTC) homes significantly influences residents’ functioning (e.g., wayfinding, self-care, and social interaction) and well-being. There is limited research on the characteristics of the built environment of LTC and its influence on residents’ quality of life in countries in the Asia–Pacific region (e.g., East Asia and South Asia). The older adult population in this region is expected to increase significantly in the coming decades. There are distinctive perceptions of nursing home, nursing home environments, and sociocultural norms in this geographic region. Given this context, a better understanding of the built environment of LTC facilities in this region can inform design professionals and policymakers for evidence-based decision-making. The present study undertakes a scoping review of the empirical research on the characteristics and influence of the built environment of LTC facilities on residents’ quality of life in the Asia–Pacific context.

**Research Design and Methods:**

Online relevant databases were used to identify articles published 2000–2021, from which we selected 33 publications.

**Results:**

Three substantive themes were generated from the synthesis of the selected publications. These themes are (a) perceptions of nursing home, (b) impact of the built environment on residents’ quality of life, and (c) assessment of the LTC built environment.

**Discussion and Implications:**

We identified research gaps in understanding the role of the built environment in nursing homes in the particular geographic context and future research directions. Five planning and design principles for LTC were derived from the synthesis of key findings to inform design professionals and policymakers.

**Translational Significance:** Lack of research on the empirical research on the characteristics and influence of the built environment of long-term care (LTC) facilities on residents’ quality of life in the Asia–Pacific context. Three substantive themes were generated from the synthesis of 33 selected publications. Five planning and design principles for LTC were derived from the synthesis of key findings. These principles will inform evidence-based decision-making by design professionals and policymakers for the development of LTC facilities in the Asia–Pacific region.

The older adult population has grown rapidly in recent decades due to the decrease in fertility, increase in life expectancy and changes in patterns of marriage (Population Division, United Nations, 2019a). It is estimated that more than 15.9% (1.55 billion) of the world’s population will be more than 65 in 2050, up from 9.1% (693.2 million) today (Population Division, United Nations, 2019b). Asia’s older adult population is projected to reach 17.5% (922.7 million) of the region’s total population by 2050, increased from 4.1% (57.6 million) in 1950 (Menon & Melendez-Nakamura, 2009). In 1950, Asia accounted for 44.0% of the global older population, but by 2050, this proportion is expected to reach 61.6% (Population Division, United Nations, 2019b), making Asia “the oldest region in the world” (Menon & Melendez-Nakamura, 2009). In China, the world’s fastest-aging country, for example, there were 191 million older adults aged 65 and over in 2020, accounting for 13.5% of the total population, and this is expected to surge to 20% by 2040 (National Bureau of Statistics of China, 2021).

The growing aging population is expected to have a multidimensional affect on society in many Asia–Pacific countries (Kim et al., 2020; Lee, 2021). Institutional long-term care (LTC) is a major area in health care policy, planning, and implementation for older adults needing ongoing personal and nursing care. The 85 years and older segment, with an associated higher level of need for care and support, places a significant demand on LTC services. The increasing involvement of women in the labor force and geographical separation from adult children have undermined the availability of informal family caregivers providing ongoing care (Gu et al., 2007; Ng et al., 2002). Additionally, the challenges in community care services and the development of diverse care options with healthy lifestyle also call for the utilization of diverse forms of congregate living settings (Lee et al., 2009; Zhan, 2002; Zhan et al., 2008). For instance, assisted living and small-group homes are innovative congregate care and support environments that aim to meet the diverse needs of older adults. However, these options remain limited in many Asian countries; hence, increasing numbers of older adults and their families in Asia feel the necessity to utilize traditional LTC in recent years (Tsai & Tsai, 2008; Tse, 2007).

LTC facilities is a generic term to describe various forms of residential LTC provision (Lee et al., 2009). For instance, in the United States, assisted living facilities provide basic care for older adults with higher self-care capacity, while nursing homes are provided for more dependent older adults. In the Asia–Pacific context, residential care facilities (RCFs) are the common form of LTC service provided for older adults, which provide different forms of LTC to meet the older adults’ physical, emotional, and environmental needs (Cheng et al., 2011; Wong et al., 2014). In China, a few of these are retirement homes (i.e., congregate living with social programs only; Chu & Chi, 2008), a few are assisted living facilities, while others provide 24/7 nursing care (Zhan et al., 2006). In this study, we use the term “nursing home” to refer to long-term RCFs, which provide accommodation, 24/7 nursing, and personal care to older adults.

The built environment of an LTC setting includes the geographic location, architectural or spatial layout, and microenvironmental features (e.g., lighting, ventilation, and furniture configuration). The quality of the built environmental features in a nursing home significantly influences the quality of life (QoL) of older residents, and persons with dementia in particular (Lee et al., 2009). An appropriately designed built environmental setting can have a critical role in contributing to the quality of care provided and QoL of the residents (Alzheimer Disease International, 2015; Fleming & Purandare, 2010). In the context of the coronavirus disease 2019 (COVID-19) pandemic, the built environment of nursing homes has become even more important. Older groups, especially those in nursing homes, have more vulnerable immune systems and are more susceptible to infection (Morley & Vellas, 2020). The typical need for close physical contact between older adults and caregivers makes it unlikely to keep older adults in quarantine on their own, which increases the risk of cross-infection (Wang, 2021). The situation could be worse in Asian nursing homes, which are often associated with a compact living environment and higher room occupancy (Tao et al., 2018). Additionally, older adults are likely to experience greater social isolation under stringent infection control measures, where they are not able to have family visitors or access to social software such as FaceTime or WeChat due to the “digital divide” caused by general cognitive decline and low Internet skills and literacy of older adults, social ageism, and the complexity of smartphone use, especially for those with dementia (Friemel, 2016; McDonough, 2016).

There is limited research on the quality of the LTC built environment and its affect on residents’ QoL in countries in Asia. Compared with nursing homes in a Western context, nursing homes in Asia are often government-run with a higher number of beds and higher occupancy rates (Chu & Chi, 2008; Cheng et al., 2011). In general, they tend to be smaller in size, with a higher number of people sharing the same bedroom, and more institutional physical environment, with a fewer number of social spaces and less variety of activities (Chang, 2013; Cheng et al., 2011; Tsai & Tsai, 2008; Tse, 2007). Given that Asian countries have distinctive features of a nursing home environment, overall perceptions of nursing homes, and sociocultural norms, there is a need for a better understanding of the built environment of nursing homes and its influence on residents’ QoL in countries in the region. For these reasons, a scoping review was conducted in order to systematically map the research done in this area, as well as to identify any existing gaps in knowledge. The objective of this scoping review is to identify and synthesize empirical research on the characteristics and influence of the built environment of LTC facilities on residents’ QoL in the Asia–Pacific context. The following research questions were formulated: What characteristics of the nursing homes’ built environment have been examined in the Asia–Pacific region? What are their influences on residents’ QoL? The findings of this article will inform public policies for LTC, planning strategies, and design guidelines for the development of built environment of care settings more supportive of the residents’ functioning and well-being.

## Method

**Figure 1.**
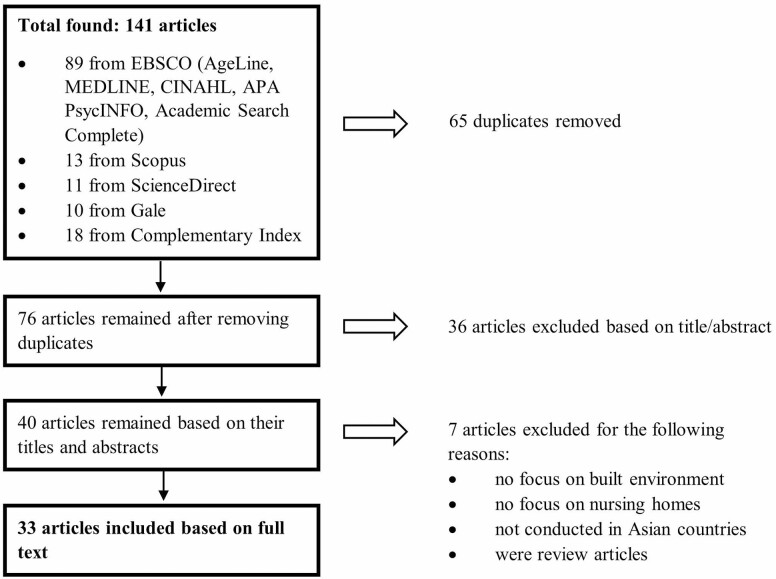
Systematic search and selection process.

This study follows Arksey and O’Malley’s (2005) five steps scoping review framework: (a) confirming the primary research question; (b) identifying the related studies through a systematic search; (c) study selection; (d) extracting key information and creating a data-charting form; and (e) collating, summarizing, and presenting the results (Figure 1). Having identified the research questions, a systematic search of related articles was conducted. The following bibliographic databases were accessed in July 2021: AgeLine, MEDLINE, Cumulative Index to Nursing and Allied Health Literature (CINAHL), APA PsycInfo, ScienceDirect, Academic Search Complete, Scopus, Gale, and Complementary Index. The initial search included broad concepts introducing “environment,” “older adults,” “Nursing home,” and “Asia Pacific.” Specific search terms have been then supplemented to narrow down the results and increase relevance:

(1) (physical environment OR built environment OR outdoor environment) AND
(2) (nursing homes OR LTC OR care facilities OR RCFs) AND
(3) (Asia–Pacific OR Asia OR China OR Japan OR Hong Kong OR Singapore OR Korea)

Related words and equivalent subjects were applied in the search for increased relevance. A manual search was also launched on the reference lists to identify related articles. The results were limited to (a) peer-reviewed articles, (b) English publications, and (c) published during 2000–2021.

Gray literature, book chapters, conference papers, and news coverage were not included in the present study. Also, we excluded articles if the study: (a) focus did not include the built environment, (b) focused on health care settings other than nursing homes, and (c) was not conducted in an Asian country. The search yielded 141 articles in total and after removing duplicates, 76 items remained. The abstracts of those items were reviewed, and 36 articles were excluded as those studies did not meet the inclusion criteria. Further screening of the full text of the 40 remaining articles was conducted, whose eligibility was then confirmed, and 33 articles that indicated explicit inclusion or mention of the built environment were included in the final review. Given that a scoping review is not necessarily intended to assess the quality of the studies, the articles included in the present study were not selected or weighted on the quality of the evidence provided (Arksey & O’Malley, 2005).

A data-charting form was developed by both reviewers (coauthors) to determine which variables to extract. The two reviewers independently charted the data, discussed the results, and continuously updated the data-charting form in an iterative process. We abstracted data on country of origin, study focus, method (e.g., sample size and age), and key findings (e.g., built environmental features, residents’ QoL, and implications for physical environment). Analysis was conducted in compliance with the fourth stage of the scoping review framework, where key information about the selected articles was extracted. Information about the study setting, focus, methods, sample characteristics, major findings, and discussions were synthesized and entered in a data-chart form (Table 1). Key findings from the studies were compared for similarities and differences. We identified the limitations of each study and their implications on planning strategies, design guidelines, and policies for built environment of nursing homes. The thematic synthesis of evidence from the identified articles and gaps based on the information extracted concluded the final stage of the review.

**Table 1.** Summary of the Reviewed Articles (*n* = 33)

Study; setting	Type of study; focus	Methods; age (if applicable)	Key findings	Discussion; limitation	Built environment implication
Chang (2013); Gyunggido, Korea	Individual level; experiences of life in nursing homes	Ethnographic (*n* = 11 aged residents); 76–96	Confirming positive and negative emotions that older adults typically experience while in nursing homes Residents perceive nursing homes as facilities, not homes. Older adults who will spend the rest of their lives in facilities may not be ready to accept the situation	The essence of the positive and negative feelings; residents with cognitive impairments or language deficits were excluded and residents of other types of care facilities need to be included	Planners and nurses should try to provide a home-like built environment within nursing homes in order to help residents experience better emotions
Cheng et al. (2011); Beijing, China	Individual level; physical and social environment of RCFs and well-being of older adults	Ethnographic (*n* = 46 older adults/family members/facility managers); 60s–90s	Each RCF, as a place with its unique physical and social environment, has a significant influence on the older adults’ physical and psychological well-being as well as the quality of care	Multiperspective analysis of the relationship between health and place in RCFs; older adults and family members interviewed are not matched within families	Designers should consider creating therapeutic landscapes and a sense of place
Cheng et al. (2012); Beijing, China	Individual level; multidimensional access to residential care and motivations of choosing a RCF	Ethnographic (*n* = 46 older adults/family members/facility managers); 60s–90s	Geographical access, information access, economic access, sociocultural access, and sociomanagerial environment are five significant factors influencing older persons’ choices of a specific RCF	Suggestions on improving the accessibility of residential care for older people; homogeneity of the sample	Physical environment and amenities of RCFs at the micro-level, such as private rooms with telephones, green lands, and bathrooms can make a difference to older adults’ choices
Chuang and Abbey (2009); Taiwan, China	Individual level; culture of nursing home and life experience	Ethnographic, including long-term observation (*n* = 60 older residents/staffs)	A tedious, monotonous, idle, and lonely life is experienced by the residents, and insufficient staffing is obvious, despite the legal staffing requirements being met. The residents, whether consciously or not, consider themselves to be the patients of a hospital	Exploring factors that contribute to the tedious nursing home culture; different types of aged-care facility need to be examined	LTC facilities need to change their hospital-based layout, remove their traditional hospital-like image and work towards more home-like settings
Hsieh et al. (2012a); Taiwan, China	Individual level; residential quality of institutional public spaces for aged residents	Content analysis and statistical association (*n* = 10 panel members)	Indoor environmental quality, safety equipment for the prevention and management of disasters occurring in residents’ daily lives, provision for social interaction space are important factors for protecting residents’ physical and psychological well-being	Comparison with other research results in different cultural context. Gap found between this study and the existing literature; did not include opinions from architects, policymakers, or residents	Planners may consider improving indoor environmental quality (e.g., proper temperature, natural lighting, and good ventilation) and providing more interaction space
Hsieh et al. (2012b); Taiwan, China.	Individual level; indoor environment (IE) quality and ease of administration	Ethnographic (*n* = 115 facility managers)	The levels of the LTC institution managers’ perceptions of the importance of indoor environment indicators differ from the levels of difficulty in implementing them. Ventilation is perceived as the most important IE factor, while temperature and humidity factors are most difficult to implement	Exploring ways to improve IE quality; confounding factors that may influence IE quality were not contained	Indoor environment, including temperature, humidity, natural lighting, lighting, ventilation, and sound, should be attached more importance
Hwang et al. (2013); Taiwan, China	Individual level; quality of care and life experience	Ethnographic (*n* = 12 older adults); 65–94	Three themes of caring perceived by older adults are: calming the body, respectful communication, and enriching life. A care model synthesizing the themes has been developed to understand the nature of care	Different perceptions of care in different context; subthemes are interrelated and not mutually exclusive	Physical configuration should provide an atmosphere that does not make older adults feel like patients
Kasai et al. (2015); Japan	Individual level; group homes, built environment and behavioral problems	Statistical association, including observation (*n* = 74 older adults with dementia); average 81	Group homes run by the LTC insurance system appear to be effective in improving environmental factors for moderate AD patients and reducing BPSD, including aggressiveness, affective disturbances, and anxieties and phobias	Assessing the results and exploring the affects of group homes on behavioral problems related to dementia; the assessment of BPSD symptoms is not so accurate	Small unit size and individual rooms can provide a better environment for elders with dementia
Komatsu et al. (2007); Aichi, Japan	Individual level; relocation to health care facility and changes in environment	Ethnographic, including observation (*n* = 8 older adults); ≥65	Background and personal factors, relocation-related factors and physical and social environmental factors can influence the outcome of relocation to HCFs through the affects of cognitive appraisal, adaptive tasks, and coping skills	How to understand the relocated elders and the kind of environmental changes to which elders adapt using a conceptual model; small sample size (*n*)	Noninstitutional environment is of significance for elders to relocate to HCFs
Kwong and Kwan (2002); Hong Kong, China	Policy level; quality of care, physical environment, and developments of private residential care homes	Simple content analysis	These problems remain in private residential care homes: the existence of substandard private aged-care homes without a license, the provision of substandard “places” to older adults, ineffective inspection, a lack of grading to indicate the quality and a general neglect of the quality of care	Recommendations to address those remaining problems and concerns of residential care homes; lack of specific sample or case	The physical environment as well as the quality of care of private residential care homes should be regulated, monitored, and graded
Lee (2001); Hong Kong, China	Individual level; adjusting experiences of relocation to care home	Ethnographic (*n* = 18 older adults); 70–86	Many adjusting experiences suggested in the literature are not regarded as important by Chinese elders Establishing relations with other residents and staff appears to be a particular challenge in the Chinese context	Comparisons with other studies in different sociocultural context; small sample size (*n*)	Built environment that facilitates communication among residents and between residents and staff is needed to help the process of relocation
Lee et al. (2013); Korea	Policy level; care environment design and holistic health	Directed content analysis	Various construction or design matters must be considered during spatial planning to promote holistic health. The placement plan is the most important element among the five construction design elements	Plans for future study; other types of guidelines need to be examined	Planners should extract key components from mountainous design guidelines and pay more attention to important aspects of care environment to improve the QoL of residents
Lee et al. (2017); Australia and Korea	Building level; properties of architectural plans and potential influencing factors	Comparative case study using computational approaches (*n* = 6 aged-care facilities)	Syntactical and cultural characteristics can be observed in the aged-care settings, thus reflecting the residents’ sociocultural preferences or needs	Exploring possible reasons for international differences in designs of age care facilities; small sample size (*n*)	Spatial configuration of aged-care homes should be more consistent with social and cultural cognition
Low et al. (2007); Hong Kong, China	Individual level; perceptions of privacy and life experience	Ethnographic (*n* = 20 older residents); 65–91	Many Chinese older people perceive privacy as “not necessary” in residential care homes and have lowered expectations for individual private living	Comparison with the existing literature (e.g., on perceptions of privacy in western context); lack of comparison with residents from other local private care homes	Privacy can be supported by adequate physical layout and practice, among which bedroom is underlined
Lu and Wang (2014); Hong Kong, China	Building level; characteristics of built environment of care units and comparison with their counterparts	Descriptive analysis, case study (*n* = 25 units in public hospital in China)	There are dramatic differences between the spatial characteristics of acute care units in China and the United States, including unit configuration, percentage of private rooms, carpeting, visibility, as well as distance to supplies and charting	Analysis of the primary forces shaping those physical designs; small sample size; the analysis is limited to the analysis of unit floor plans	Planners should consider reducing the long walking distance from nursing station to patient bedside and improving the visibility to beds from a nursing station
Sawamura et al. (2013); metropolitan areas, Japan	Individual level; principles of care provision and building structures	Statistical association (*n* = 387 individuals)	The structure of unit-care model facilities (i.e., dividing a large-scale nursing home into multiple small care units) is undoubtedly advantageous for providing individualized care	The influence mechanism of building structure on principles of care provision and how governmental requirements can help; observational studies are needed to confirm the results of survey	Planner and designers may consider implementing the unit-care model when building a nursing home
Song et al. (2018); Jinan, China	Individual level; challenges of daily activities in LTC facilities	Ethnographic (*n* = 5); 70s–90s	Five themes that related to challenges emerged: physical environment, staff care, care from family members, coresidents in the facility, and resident-developed strategies	Exploring factors that induced challenges in LTC facilities and ways to address them (in relation to other research); small sample size (*n*) and lack of observational data	Modify the LTC facility environment to improve the quality of care Improve the wayfinding performance and try to create a home-like environment
Sun and Fleming (2021); Singapore	Individual level; development of assessment tool for built environment of nursing homes	Statistical association (*n* = 22 participants using the tool)	The Singapore Environmental Assessment Tool (SEAT) has a high level of usability and combines a scale with an acceptable level of reliability and validity with items that have been included to foster systematic discussion, evaluation, and education for people engaged in the development of facilities for people with dementia	Opinions of users. Strategies of improving the SEAT. Plans for future research; more information, education, and training are needed for participants	Cultural sensitives should be taking into consideration when assessing built environment Stakeholders may need to improve understanding of dementia-enabling environments
Tao et al. (2018); Hong Kong, China	Individual level; floor plans’ legibility and wayfinding satisfaction	Statistical association (*n* = 181 occupants); ≥70	Increasing complexity in floor plans negatively affects residents’ wayfinding ability and thus their satisfaction	Plans for future research. Explanations of the results in context; lack of analysis of vertical circulation	It is imperative for architects to design clear circulation patterns in care and attention homes so as to improve wayfinding performance
Tao et al. (2018); Hong Kong, China	Individual level; bedroom privacy and well-being and compact living environment	Statistical association (*n* = 231 residents in care homes); ≥70	The crowded living environment in aged-care facilities may compromise the well-being of residents. These architectural parameters are influential predictors for bedroom privacy in relation to older adults’ well-being: total open surface per unit, openness/solid ratio per bed, height of partition wall, number of people per unit, and personal control over bedroom privacy	How built environment features can have an influence on privacy and well-being of residents; analysis of affects of cultural context on the perception and need of privacy is needed	Renovation or new design of bedrooms in nursing homes should pay attention to the five parameters to provide residents with adequate personal space The provision of privacy for older adults should be balanced with their needs for social interactions and open area
Traphagan and Nagasawa (2008); Japan	Policy level; dementia care and development of group homes	Simple content analysis and case study (*n* = 1 new group home)	The LTC insurance program has stimulated the creation of new contexts of caring for dementia sufferers in Japan, including group homes such as those at Zenjinkai that attempt to create a home-like environment for the residents	Practical and operational problems of the new forms of care homes; small sample size (*n*)	A home-like environment within care homes is helpful to better relationships between staff and residents and is significant to the life experience of residents with dementia
Tsai and Tsai (2008); Taiwan, China	Individual level; life experience of elders in nursing homes	Ethnographic, including observation (*n* = 33 older residents); 65–97	Life in a Taiwan nursing home is perceived as too highly structured. The core theme of older participants’ lived experience in the nursing home was a temporary home to nurture their health. They perceived that they had been relocated from their “real homes” for health reasons, just as people are sent to the hospital when they are sick	Assessing the result in relation to similar studies in different cultural context; the participants were referred by nursing home staff	Planners should strive to provide a variety of spaces so as to provide a variety of activities and enrich older residents’ life experience
Tse (2007); Hong Kong, China	Individual level; perceptions of nursing homes and strategies for improvement	Ethnographic (*n* = 118 older persons); 60–89	Most elders’ nursing home-related beliefs are found to be negative, while some elders think positively of nursing home. Nursing homes are perceived to fill up with sick people and strangers. The living environment, hygiene and even the training and the quality of nursing home staff were considered as inadequate and unfriendly	Comparison with the outcomes of studies in western context. Exploring sociocultural factors; variety and representativeness of sample	Living environment of care homes needs to be improved, modernized, and beautified to change the image in the eyes of older adults
Tsuchiya-Ito et al. (2019); the western and central part of Japan	Individual level; physical home environment and subjective well-being	Statistical association (*n* = 1,928 individuals); ≥65	Issues related to safety, health, and amenity of the physical housing environment are associated with negative aspects of health and most pronounced among those with low ADL independence	Analysis of the influence mechanism of physical environment on subjective well-being; longitudinal studies are needed and the raters might have been influenced by the clients’ health status	Planners need to consider the importance of appropriate environmental conditions (e.g., access to emergency assistance, indoor temperature, sanitary condition, etc.) for older adults
Wang (2021); China	Policy level; environmental factors essential for infection control	Simple content analysis	Proper planning and design of the built environment promote strategies for infection control in senior-living facilities	How environmental factors can help with infection control; lack of observational data and statistical support	Infection control factors should be given priority in the design and development of environments for senior living. Factors at site, building and room level should all be taken into account
Wang et al. (2021); China	Policy level; problems and sustainable development of LTC	Simple content analysis (*n* = 70 news reports)	Twelve main problems for LTC were identified and further classed into three different categories according to their connection to different stakeholders, including service providers, government, and the public	Possible causes of each problem and ways to address them; news reports searches were conducted only for portal websites, which were retrieved by Google	Planners should consider the needs of older residents in mountainous areas to help them change their stereotypes about LTC
Wong et al. (2014); Hong Kong, China	Individual level; indoor environment and dementia and behavioral problems	Simple content analysis, including observation (*n* = 36 individuals, including dementia residents, staffs, and family members)	Among lots of indoor environment elements, the acoustic environment, lighting, and thermal environment are the most important factors affecting the physical and psychosocial performance of older residents with dementia	Exploring how indoor environment factors can have an influence on the experiences of behavioral problems of dementia residents; small sample size	For dementia-specific RCFs, careful scrutinization of both indoor environment design and external surrounding environment is needed
Wu et al. (2008); Hubei and Shanghai, China	Policy level; delivery and financing mechanisms for institutional care for elders	Simple content analysis, including on-site observation (*n* = 12 nursing homes)	In terms of institutional care, welfare institutes and homes for the aged have played the most important role in providing LTC for rural elders. These organizations have undergone rapid changes in recent years and there are still a lot of issues to focus on	Policy implications and plan for future study; policy proposals recommended lack data support	Planner should consider the needs of older adults in rural areas as well as their urban counterparts
Wu et al. (2009); Taiwan, China	Individual level; phenomenon of nursing home care and family choice	Ethnographic, including observation (*n* = 60 older residents/family members); ≥65	Nursing home care for older people in Taiwan is understood to be a process of forced choice, involving three stages; namely, “becoming a problem,” “making a forced choice,” and “coping with the forced choice”	Exploring cultural and social factors influencing the phenomenon of care of older people; representativeness of the sample	Planners should try to create a care environment that is easy to accept for the relocated residents
Xuan et al. (2019); China	Individual level; unit layout and nurse experiences	Ethnographic, including on-site observations (*n* = 29 nurses)	The clinical workspaces ranked by importance were patient rooms, NS, workstation on wheels (WoWs), medication room, physician’s office, disposal room, examining room, and back corridor. The frequency of links between the NS and medication room, medication room and patient room, and patient room and WoWs was very high	Comparison with other research in western context. Plan for future study; more studies are needed to examine different unit types	Corridors in nursing units should be perceived as workspace and communication hub, which should be better designed
Xuan et al. (2020); China	Individual level; visibility, proximity and communication, privacy, and efficiency	Statistical association, including observation (*n* = 70 nurses)	Visibility and proximity have a substantial influence on nurse communication patterns, perception of privacy, and efficiency of care giving in double-corridor nursing units	How designs of nursing unit may have affects on visibility and proximity and thus influence communication, privacy, and efficiency; results limit the broad generalizability to other types of nursing units	A geographically contiguous layout could enhance work efficiency. High visibility enhances communication and reduces the time needed to locate team members
Yang et al. (2020); Hong Kong, China	Individual level; privacy in physical environment and social interaction	Ethnographic (*n* = 50 older adults); ≥65	Privacy can be of significance in facilitating social interaction among older adults. The more privacy there is, the less mobility and more formal interaction there will be	Comparisons with other studies using traditional ethnographic methods (e.g., observations and interviews). Directions for future research; the behavior of care staff missed, and absolute numbers of locations are not available	Designers should pay more attention to the quality of and privacy perceived in bedrooms. Transitional spaces between private and public areas are important
Yu et al. (2017); Anhui, China	Individual level; built environment and care service and QoL in rural context	Statistical association (*n* = 242 residents); 60s–90s	Rural older adults’ QoL can be accurately predicted by four built environment factors, including room distance, space, barrier-free design, indoor environment, and two care service factors, including daily care services and cleaning services	Further explanation of the positive relationships between environmental factors and care services factors of rural nursing homes and older adults’ QoL; lack of observational data	Designers of rural nursing homes should pay close attention to the design features of the built environment, for example, the distances between the rooms that support the activities of daily living for older adults should be shorter

*Notes:* RCFs = residencial care facilities; LTC = long-term care; ADL = activities of daily living; NS = nurse station; QoL = quality of life; AD = Alzheimer’s disease; BPSD = behaviour and psychological symptoms of dementia; HCFs = health care facilities.

## Results

As the largest developing country and the fastest-aging country, China seemed to draw the most attention, with the bulk of the included studies (21 of 33, 64%) conducted in that country, including Special Administrative Regions Hong Kong and Taiwan. The rest of the studies were conducted in developed economies, among which Japan ranked first (six of 33, 18%), followed by Korea (four of 33, 12%), and finally Singapore (two of 33, 6%). Although low-income countries in Asia, such as Thailand, India, and Iran, also have a growing aging population (Population Division, United Nations, 2019b), the research gaps in these geographic contexts are still notable.

Most studies were carried out at the individual level (25 of 33, 76%), while others were at the policy level (six of 33, 18%) or building level (two of 33, 6%). Qualitative research methods were commonly employed, with interviews, focus groups, and on-site observations being the most popular (16 of 33, 48%). A few studies used content analysis and occasionally employed case study methods (nine of 33, 27%). The remaining studies involved quantitative methods, with statistical associations (nine of 33, 27%), and mixed methods were occasionally adopted. Two studies (6%) employed innovative methods, that is, visibility graph analysis and isovist analysis (Lee et al., 2017) and Bluetooth Low Energy indoor positioning systems (Yang et al., 2020).

Despite the diversity of research methods and geographic settings, the characteristics of the samples tended to be similar. The study participants were predominantly older residents of nursing homes aged 60 and older. A few studies included older community-dwellers for the collection of viewpoints from different perspectives. Caregivers, family members, and the managements were also frequently involved as supplementary data sources (Hsieh et al., 2012b; Xuan et al., 2019, 2020). Older adults with advanced dementia or severe mental illness were commonly excluded, due to challenges in collecting their QoL-related data directly from them (Chang, 2013; Kasai et al., 2015; Komatsu et al., 2007; Tsai & Tsai, 2008). The majority of the studies were conducted in urban or suburban contexts. Only three out of the 34 selected articles were focused on rural settings (Hwang et al., 2013; Wu et al., 2008; Yu et al., 2017). Three substantive themes were generated from the synthesis of the reviewed literature (a) perceptions of nursing home, (b) impact of the built environment on residents’ QoL, and (c) assessment of the nursing home built environment.

## Perceptions of Nursing Home

In general, the traditional image of a nursing home environment (i.e., institutional, less private, unsupportive, and inadequate) was prevalent among Asian older adults (Chang, 2013; Chuang & Abbey, 2009; Hwang et al., 2013; Low et al., 2007; Tsai & Tsai, 2008; Tse, 2007). Older adult residents residing in LTC facilities and their family members in Asia tended to think of nursing homes environment as hospital-like, which was more of a “facility for patients” than a “second home or place to enjoy the elderly lives” (Chang, 2013; Cheng et al., 2011; Chuang & Abbey, 2009; Tsai & Tsai, 2008; Tse, 2007). Environmental features such as spatial layout, room arrangement, and decoration were considered as unsatisfactory; these were perceived as making the older adults feel like patients (Chang, 2013; Tsai & Tsai, 2008). In addition, the residents often felt that their privacy would be undermined by inadequate and inconsiderate physical layout (Low et al., 2007; Tse, 2007). Older residents were living in compact and crowded environments due to high occupancy rates and the collectivist design trends, where they were exposed to not only other residents with different health conditions, but also to several staff, visitors, and personal assistants (Chuang & Abbey, 2009; Low et al., 2007). The resident bedrooms did not generally support privacy, such as no locks for the room entrance, absence of partitions or screens between the beds, and a lack of transitional space between private and public areas (Tao et al., 2018; Tse, 2007; Yang et al., 2020). However, general expectations and needs for privacy were relatively low among the residents in the context of Asian culture (Low et al., 2007), while there is evidence that an individual private room was a preference (Tao et al., 2018; Yang et al., 2020). A few studies indicated that some residents found the built environment unsupportive for activities of daily life (Chuang & Abbey, 2009; Song et al., 2018; Tse, 2007; Yu et al., 2017). The residents, especially those with dementia or physical disability, encountered obstacles in daily activities and called for barrier-free designs (Kasai et al., 2015; Song et al., 2018), which indicated that the environment of the setting did not compensate for their impaired mobility or cognition. Besides, there was a general perception that the environment was tedious, and lifeless, and made them less energetic (Chuang & Abbey, 2009).

Nevertheless, a few older adults and their families had positive perceptions of the nursing home environment, which provided higher safety, quicker access to emergency help, and greater social participation (Chang, 2013; Tse, 2007). Apart from economic and sociocultural considerations, the physical environment and facility management were important determinants for older adults and their families when choosing a specific RCF (Cheng et al., 2012; Wu et al., 2009). It was notable that older residents in rural settings hold similar perceptions of nursing home environment as their urban counterpart, namely monotonous, inadequate, and institutional (Hwang et al., 2013).

A few studies have looked specifically at the experiences and perceptions of nurses and managers. Xuan et al. (2019) suggested that the clinical workspaces perceived by nurses were the patient room, nursing station, workstation on wheels, medication room, physician’s office, disposal room, examining room, and back corridor. Corridors in nursing units were often perceived as a workspace and communication hub, which were considered as not well designed in terms of length, width, turns, and seating. Hsieh et al. (2012b) examined LTC managers’ perceptions of indoor environment indicators and found ventilation and lighting weighed the most. Although managers were aware of the importance of the indoor environmental factors (i.e., temperature, lighting, ventilation, sound, and humidity), it was difficult to improve indoor environmental quality through management and it needed the help of proper planning and design.

## Impact of Built Environment on Residents’ QoL

Overall, the built environmental features of LTC facilities could have a significant influence on QoL of older residents through complex mechanisms, mainly reflected in the impact on quality and efficiency of care and the resident’s perceived autonomy and individuality (Lee et al., 2009; Sawamura et al., 2013; Tsuchiya-Ito et al., 2019; Wang et al., 2021; Xuan et al., 2020). In terms of overall spatial layout, it was indicated that the scale or size of the units in the facility could be smaller. A large nursing home divided into multiple small care units could promote individualized care with close proximity to clusters of resident rooms and nurses’ closer assessment of residents’ status (Sawamura et al., 2013), and potentially reduce behavioral and psychosocial symptoms of dementia. For example, older adults with dementia in small nursing homes in Japan have significantly fewer symptoms than their counterparts in communities or large nursing homes, such as aggressiveness (29% less, *p* = .035) and anxiety and phobias (42% less, *p* = .001) (Kasai et al., 2015). The complexity of the floor plan was found to undermine the wayfinding ability of the residents (Tao et al., 2018). Besides, from the perspective of holistic health, general planning (e.g., location/arrangement of amenities), spatial layout (e.g., spatial adjacencies, room size, and configuration), and internal design (e.g., furniture, lighting, and temperature) could stimulate healthy behavioral patterns and promote total health (i.e., physical, mental, and social health) for older residents (Lee et al., 2013).

In terms of microenvironmental features, it was generally recognized that a home-like environment (e.g., objects associated with personal memories and residential furniture) was of significant benefit to various aspects of the well-being of older adult residents (Chang, 2013; Hwang et al., 2013; Komatsu et al., 2007; Song et al., 2018; Tao et al. 2018; Traphagan & Nagasawa, 2008; Wu et al., 2009). Natural landscapes and open spaces such as water and gardens were reported to have healing functions, which could provide the residents with comfort and facilitate social interaction (Cheng et al., 2011; Hsieh et al., 2012a). Indoor environmental factors also played a significant role in the well-being of the residents (Wong et al., 2014; Hsieh et al., 2012b); for instance, indoor lighting enhanced safety and mobility for the older adults, and indoor temperature and ventilation improved their comfort (Yu et al., 2017). The acoustic environment was also considered crucial for the functioning of older residents with dementia, as high noise levels in social spaces were perceived as contributing to anxiety and confusion (Wong et al., 2014). Moreover, environmental factors assisted older adults in the adjustment to relocation by helping with adaptive tasks (Song et al., 2018; Wu et al., 2009), cognitive appraisal (Komatsu et al., 2007), and improving social participation and communication with staff (Lee, 2001). Microenvironmental parameters such as “total open surface per unit” and “height of partition wall” helped with older adults’ control over privacy and gave them a sense of ownership (Tao et al., 2018; Yang et al., 2020). Bedroom privacy measured by these parameters was further found influencing older adults’ physical and mental health status according to regression modeling of data from 213 respondents in eight nursing homes in Hong Kong (Tao et al., 2018).

It was interesting to note that social interaction could be promoted in environments with more privacy, potentially related to increased one-on-one interactions in private rooms (Yang et al., 2020). Challenges in three aspects of the physical environment, including safety (e.g., access to assistance and prevention of fall incidents), amenity (i.e., the state of home disrepair), and health (e.g., thermal environment and sanitary condition), were associated with poor QoL, especially for those with low activities of daily living independence (Hsieh et al., 2012a; Tsuchiya-Ito et al., 2019). Based on multiple regression of data from 242 questionnaires, the QoL of older adults in rural settings was highly associated with four built environment factors: room distance from social spaces, room size, barrier-free design, and indoor environment (Yu et al., 2017). From the nurses’ perspective, it was found that visibility and proximity, which largely depended on environmental factors such as the type of walls and distance between workplaces, could affect communication, perceptions of privacy, and efficiency of caring greatly (Xuan et al., 2020). For instance, it is estimated that 28% of communication would be reduced by low visibility among adjacent spaces in the environment (Xuan et al., 2020).

In the context of the recent epidemic, proper planning, and design of RCF at site, building, and room level (e.g., reception room for visitors and rooms with dedicated toilets) were advantageous for infection control, which could be achieved through three stages, namely keeping COVID-19 from entering, preventing spread, managing infection, and illness (Wang, 2021).

## Assessment of the Built Environment of Nursing Home

Findings under this theme mainly focused on environmental assessment tools and development of LTC facilities as well as the existing problems of their environment. Content analysis and case study were the most common methods, while quantitative methods were involved in the literature concerning assessment tools. It was suggested that the provision of substandard “places” to the residents, along with other nonstandard phenomena (e.g., ineffective inspection, existence of substandard nursing homes without a license, and lack of qualified LTC professionals), remained a serious problem in contemporary private RCF and an obstacle for development, compromising the resident’s QoL (Kwong & Kwan, 2002; Wang et al., 2021). Appropriate planning, policies, and systems could be key to addressing these problems and contributing to the development of the nursing home environment (Hsieh et al., 2012b; Traphagan & Nagasawa, 2008; Wang et al., 2021), especially in rural settings (Wu et al., 2008).

On the other hand, cultural reflection in the environmental features was found in nursing home settings through comparative case studies (Lee et al., 2017; Lu & Wang, 2014), indicating that built environment reflected the residents’ sociocultural preferences or needs (Chuang & Abbey, 2009; Lee et al., 2017; Sun & Fleming, 2021). For instance, there are notable differences between built environment characteristics of nursing units in China and the United States, including physical configuration, room occupancy, and quality of social spaces (Lu & Wang, 2014). As for assessment tools, Sun and Fleming (2021) illustrated that the Singapore Environmental Assessment Tool (SEAT) had a high level of usability for aged-care facilities and would be more reliable and valid if users were familiar with dementia-enabling environments in the Asian context.

## Discussion

In this scoping review, we identified 33 primary studies addressing research evidence on the built environment of nursing homes in the Asia–Pacific region published between 2000 and 2021. It was evident that the general beliefs for nursing home environment among Asian older adults were generally negative, and environmental features at different scales had a significant influence on the physical and mental well-being of older residents. The findings of this review are consistent with the work of Sun and Fleming (2018), which explores the characteristics of the built environment for people with dementia in East and Southeast Asia. In the current review, the majority of the studies focused on behavioral and psychosocial aspects of the built environment of nursing homes, which relied on ethnographic methods and drew evidence from older residents’ life experiences in nursing homes, while a few studies examined specific environmental features based on statistical associations.

Compared with the previous review (Sun & Fleming, 2018), innovative methods and broader substantive focus on this topic were observed in the studies included in the present review, which brought about interesting findings and new perspectives. For instance, evidence indicates that more privacy with an increased number of private or double-occupancy rooms, more social interactions among older residents (Yang et al., 2020), and the role of the built environment of care settings in infection control in the context of COVID-19 has been examined (Wang, 2021). Research gaps remain in the field of nursing home environment in the Asian context (Alzheimer Disease International, 2015; Sun & Fleming, 2018). As mentioned previously, it was notable that little evidence was available in developing countries such as Thailand and the Philippines. This might result from the undeveloped aged-care industry in these regions, which has received less attention. Current studies are concentrated in China, Japan, Korea, and Singapore. Second, rural nursing home environments received limited attention, with most of the studies focused on urban or suburban settings. Additionally, comparative studies were rare in Asia–Pacific, not only between different regions, but also between urban and rural areas and between nursing homes of different natures (i.e., different operating models and financing mechanisms). Third, qualitative studies tended to have conclusions without on-site observational data to support the interview results (Song et al., 2018; Wang, 2021); a few studies were limited in the homogeneity of samples or had small sample sizes, making them limited in generalizability. Finally, the quantification of environmental factors such as lighting, ventilation, and visibility lacked unified and reliable standards. Statistical studies focusing on environmental features tended to have their own quantitative standard, which made the results hard to compare. The lack of reliable environmental assessment tools suitable for the sociocultural contexts of Asian countries was also notable (Sun & Fleming, 2021). Additionally, few studies have considered environmental features of nursing homes as the primary research focus, whereas most studies only considered the built environment as one of the parameters when studying other issues such as the residents’ QoL and the relocation process.

## Future Research Directions

Future research could shed more light on the built environment of the nursing homes in undeveloped regions within the Asia–Pacific region. Current findings and experience are not necessarily applicable to those countries, due to their significant social, culture, and economic differences from upper-middle-income countries, and thus targeted research is needed for the development of aged-care settings in those areas. In addition, future studies should focus more on rural settings (Wu et al., 2008; Yu et al., 2017). Older adults in rural areas tended to have different behavior patterns and preferences for environment compared to their urban counterparts (Yu et al., 2017). For example, rural residents may consider public spaces in a nursing home more important than their counterparts in the cities, because collective activities are more common in the countryside; hence, they may prefer communal rooms, while urban older residents may prefer private rooms. There is a need for more insights into rural aged-care settings to improve the rural residents’ QoL. Ideally, studies should find out perceptions of nursing homes among rural residents and obstacles they encounter in nursing home settings and identify the significant environmental features that influence their QoL.

Developing environmental assessment tools that account for Asian cultural sensitivities is identified as an important area for future research. There were few assessment tools that can provide information on the absence and presence of built environment characteristics supporting older residents in Asia, until the introduction of Singaporean version of SEAT (Sun & Fleming, 2021). However, more cultural adaptations of assessment tools are needed for other parts of Asia to provide evidence-based support and guidelines for the planning and design of LTC facilities. It would help to provide a reliable standard for quantifying environmental features as well.

More insights into how the built environment of the nursing home can help improve older adults’ QoL in the pandemic is needed. It’s evident that the built environment is an important factor to support infection control (Wang, 2021). However, beyond the morbidity resulting from the coronavirus, other aspects of the pandemic affecting older adults also need to be considered (e.g., providing space for older adults to meet visiting families, and providing a safe environment for social interaction within the nursing home).

In terms of research design, the diversity of samples could be improved in future studies. For example, residents’ lived experiences and people with different levels of dementia, and other subpopulations such as older adults with hearing problems could be engaged (Cheng et al., 2011, 2012; Kasai et al., 2015). Besides, LTCs of different organizational contexts (e.g., private, community-run, and government-run) could receive comparative examination (Lu & Wang, 2014; Traphagan & Nagasawa, 2008; Wu et al., 2008). Most existing studies have drawn evidence from government-run nursing homes. However, it’s important to note that the environment and expectations of residents could be different in other types of nursing homes. To explore how findings may differ in different broader contexts, comparative research between diverse settings (e.g., Asian, Western, low-income, developed, urban, and rural) are significant, which could provide us with a better understanding of the role of diverse environmental features in residents’ QoL.

## Implications for Planning and Design of Nursing Homes

We have identified planning and design principles relevant for nursing homes in the Asia–Pacific context based on the key findings from the empirical research examined in this review.

### Provision of Supportive Spatial Layout

Nursing homes should divide large structures into smaller care units to provide more individualized care. Workplaces such as nurses’ stations, medication rooms, and physician offices should be more concentrated and geographically contiguous, which may improve the communication among nurses and the efficiency of caring. The spatial layout needs to be legible for easier navigation, both vertical and horizontal, to support older residents’ wayfinding performance. In addition, accessibility to various social spaces (e.g., dining, activity, and lounge) and assistance should be improved through a more decentralized arrangement of these social spaces (Kasai et al., 2015; Lee et al., 2013; Lu & Wang, 2014; Tao et al., 2018; Tsuchiya-Ito et al., 2019; Xuan et al., 2019, 2020; Yu et al., 2017).

### Provision of Home-Like Environment and Sense of Familiarity and Belonging

Nursing homes need to change their traditional hospital-like image and implement a noninstitutional or home-like setting. A home-like setting can be achieved through several microenvironmental features. For instance, floors and walls can be covered in warm colors instead of “hospital white,” furniture should be in common household styles, and bedrooms can be decorated with items brought from residents’ original homes. A compact yet uncrowded layout can also help create a homely atmosphere, promoting social interaction, not only among residents but also between residents and staff (Chang, 2013; Chuang & Abbey, 2009; Hwang et al., 2013; Komatsu et al., 2007; Song et al., 2018; Tao et al., 2018; Traphagan & Nagasawa, 2008; Wu et al., 2009).

### Provision of Increased Privacy in Care Settings

Newly built nursing homes can be equipped with private bedrooms. In multigroup bedrooms, partitions should be set up reasonably. There should be transition space between personal and public areas within nursing homes, for example, between bedroom and dining room. The adequate physical layout is also of significance in supporting privacy. Nevertheless, it’s important that the provision of privacy for older adults should be balanced with their needs for suitable social spaces for planned activities and informal social interactions (Chuang & Abbey, 2009; Low et al., 2007; Tao et al., 2018; Tse, 2007; Yang et al., 2020).

### Provision of Positive Environmental Stimuli for Social Participation

Designers can be encouraged to incorporate natural elements such as water and flowers in the environment, and create multiple social spaces instead of one multipurpose space (e.g., dining room, lounge, small-group activity space, and reminiscence space) to generate diverse experiences and create various points of interests for positive engagement of the residents (Cheng et al., 2011; Lee, 2001; Tsai & Tsai, 2008; Wang et al., 2021; Wong et al., 2014).

### Reduction of Physical Barriers

Nursing homes should be designed to provide a sufficient level of lighting, both natural and artificial, which is essential for reducing barriers in older residents’ activities of daily life. The acoustic environment and thermal environment are also crucial to relieve anxiety and stress in older adults. Sound insulation should be considered in the living spaces of new or renovated nursing homes. Good ventilation could be achieved through reasonable layout and detailed design (e.g., the “trip doors” of traditional houses in Guangzhou, which are made up of a set of wooden crossbars that are equally spaced, separating the interior and exterior spaces while letting fresh air and sunlight in). Additionally, attention needs to be paid to barrier-free designs of the internal and external environment of nursing homes, such as ramps, handrails, and accessible toilets, to support older residents moving independently, especially those with physical problems (Hsieh et al., 2012a, 2012b; Tsuchiya-Ito et al., 2019; Wang et al., 2021; Wong et al., 2014).

## Limitations

We only included articles in English, which is not the official language in most Asian countries, and there may be relevant publications in other languages. It was occasionally challenging to decide whether studies are relevant to the nursing home environment due to different use of terminology.

## Conclusion

The role of the built environment in nursing homes is a substantial field of study in the Western context; however, there has been limited evidence in Asia. This scoping review examined articles relevant to this topic in Asian contexts, synthesized the key information and identified current research gaps, future research directions and implications for practice. The key findings from the current literature included three themes, namely perceptions of nursing homes, the impact of built environment on QoL and assessing nursing home environment. Ethnographic research methods were commonly employed, while a few studies focused on discrete built environmental features with statistical associations. By identifying the research gaps, we recommend future research to shed more light on rural care settings and under-developed areas in Asia–Pacific, develop reliable environmental assessment tools for countries in this region and make breakthroughs in research design, including the involvement of diverse subpopulations, new understandings of quantification of environmental features and comparative studies.

## Supplementary Material

igac045_suppl_Supplementary_Data
